# Supplementary material for: Tandem-repeat proteins introduce tuneable properties to engineered biomolecular condensates
Source: Chem Sci. 2025 May 5;16(23):10532–48. doi: 10.1039/d5sc00903k (PMC12076082; doi:10.1039/d5sc00903k)

Supplementary Information (SI) for Chemical Science.
This journal is © The Royal Society of Chemistry 2025

Supplementary Information

Tandem-repeat proteins introduce tuneable properties to engineered biomolecular condensates

Tin Long Chris Ng,[1,7], Mateo P. Hoare[1,7], M. Julia Maristany[2,7], Ellis J. Wilde[1], Tomas Sneideris[2], Jan Huertas[2,4], Belinda K. Agbetiameh[1], Mona Furukawa[1], Jerelle A. Joseph[5,6], Tuomas P.J. Knowles[2], Rosana Collepardo-Guevara[2,3,4], Laura S. Itzhaki[1,*] and Janet R. Kumita[1,*]

[1]Department of Pharmacology, University of Cambridge, Tennis Court Road, Cambridge, CB2 1PD, UK.

[2]Yusuf Hamied Department of Chemistry, University of Cambridge, Lensfield Road, Cambridge, CB2 1EW, UK.

[3]Department of Physics, University of Cambridge, Cavendish Laboratory, Cambridge, CB3 0FZ, UK.

[4]Department of Genetics, University of Cambridge, Downing Pl, Cambridge CB2 3EH, UK.

[5]Department of Chemical and Biological Engineering, Princeton University, Princeton, NJ, 08544, USA.

[6]Omenn–Darling Bioengineering Institute, Princeton University, Princeton, NJ, 08544, USA.

[7]these authors contributed equally

*To whom correspondence should be addressed. E-mail: jrk38@cam.ac.uk; lsi10@cam.ac.uk.

Supplementary Methods:

SDS-PAGE analysis of proteins

LCD2-CTPRs in purification buffer that had been previously flash frozen were thawed and incubated with FlAsH-EDT (final concentration 100 μM, 10 min, room temperature). The sample was mixed with 4X SDS-PAGE loading buffer (no added reducing agent) and loaded onto a 15% SDS-PAGE gel. Gels were run in 1X MES buffer (10 min at 80V followed by 45 min at 180V) and gels were imaged on a Biorad Gel Doc EZ Imager (Biorad Laboratories Inc, Watford UK) using the UV tray for fluorescence imaging. After imaging for FlAsH labelling, the gel was stained with Coomassie Blue and imaged using the white tray.

Circular Dichroism (CD) spectroscopy

CD measurements were conducted with a Chirascan CD spectrometer (Applied Photophysics, Leatherhead, UK) in 1 mm pathlength Precision Cells (110-QS; Hellma Analytics, Müllheim, Germany). Protein samples at 5 µM in sodium phosphate buffer were measured across 205-250 nm wavelengths. Spectra, taken at 1 nm intervals every 0.5 s, were recorded at 20°C, then 90°C, and again at 20°C. Each protein measurement was independently repeated three times, with data baseline corrected and averaged.

Gdn-HCl denaturation assay

The different GdnHCl concentrations were prepared by mixing the appropriate volumes of 50 mM sodium phosphate buffer pH 7.5, 150 mM NaCl, 7 M GdnHCl and 50 mM sodium phosphate buffer using a Hamilton Microlab ML510B. The protein concentration used was 15 µM and protein and solutions were dispensed into 96-well, half-area, black polystyrene plates (Corning) and covered with 96-well microplate aluminium sealing tape (Corning). Samples were equilibrated at 25 °C for 2 hr. Emission at 360 nm was measured using a BMG Labtech CLARIOstar® Microplate Reader. The top optic was used in precise mode for 2 cycles with an excitation wavelength of 295 nm and a dichroic PL325 nm filter at 25°C. Each measurement was performed in triplicate and three independent experiments were carried out for each protein variant unless otherwise specified. The data were fit to a two-state model, with denaturation curves fitted directly in GraphPad Prism 10 as described by Perez-Riba et al. [1].

LC3 pull-down assay

Ni-Charged MagBeads (GenScript, Oxford, UK) were incubated with 50 µM His-tagged CTPR4 protein (1 hr, RT) then washed with sodium phosphate buffer (x3). The conjugated beads were split into eight aliquots and incubated with purified LC3 at varying concentrations (1 hr, RT). These were washed with TBS-T (50 mM Tris-HCl, 150 mM NaCl, 0.1% (v/v) Tween 20, pH 7.5) (x3) and resuspended in sodium phosphate buffer. The proteins were eluted from beads with phosphate buffer with 500 mM Imidazole. Samples were run on 15% polyacrylamide gels for SDS-PAGE. The proteins were transferred onto a Immobilon-P polyvinylidene difluoride (PVDF) membrane (Merck Millipore) using a Pierce Power Blot Cassette (25 V, 1.3 A, 10 min). The membrane was blocked (5% Dried Skimmed Milk (Marvel) TBS-T, 1 hr), then incubated with a rabbit anti-LC3B primary antibody (GeneTex GTX127375, 1:1000 in 5% milk/TBS-T, ON, 4°C). The membrane was washed with TBS-T (3x 5 min) and incubated with swine anti-rabbit IgG / HRP secondary antibody (Dako P0399, 1:5000 in 5% skimmed milk/TBS-T) (1 hr). The membrane was washed with TBS-T (3x 5 min) and detected using Amersham ECL™ Western Blotting Detection Reagents (Cytiva), imaged using an Odyssey Fc Imager (LI-COR, Lincoln, NE, USA).

Keap1 recruitment to LCD2-CTPR4-Nrf2 condensates

The Keap1 Kelch domain was expressed and purified as outlined [2]. Fluorophore labelling of Keap1(100 μM) was carried out in 50 mM sodium phosphate buffer (pH 8.0) with 150 mM NaCl using AlexaFluor™ 488 N-succinimidyl ester at a molar ratio of 5:1 (dye-to-protein) and incubated (1 hr, room temperature). Excess dye was removed using Pierce Dye removal columns (Thermofisher) following the manufacturer's protocol. Co-localisation assays were performed by mixing unlabelled LCD2-RCTPR4-Nrf2 with fluorescently labelled Keap1. Unlabelled LCD2-RCTPR4-Nrf2 was diluted to 2 μM in 50 mM Tris HCl (pH 7.0), 150 mM NaCl, 100 mM urea, 0.5 mM TCEP, and Alexa488-labelled Keap1 was added (1:1 molar ratio). Solutions were imaged using a 63x oil objective with a green fluorescence channel for Alexa488 detection. The Bright field channel and green fluorescence channel were captured at a fixed exposure time to ensure consistency across samples. Representative images were processed with background subtraction and contrast adjustment for visualisation purposes. Partition coefficient determination was performed as described for the LC3 partition coefficient assay (Material and Methods).

References:

1. A. Perez-Riba and L. S. Itzhaki, A method for rapid high-throughput biophysical analysis of proteins, *Sci Rep*, 2017, 7, 9071.
2. S. K. Madden, A. Perez-Riba and L. S. Itzhaki, Exploring new strategies for grafting binding peptides onto protein loops using a consensus-designed tetratricopeptide repeat scaffold, *Protein Sci*, 2019, 28, 738-745.

Supplementary Figures and Tables:

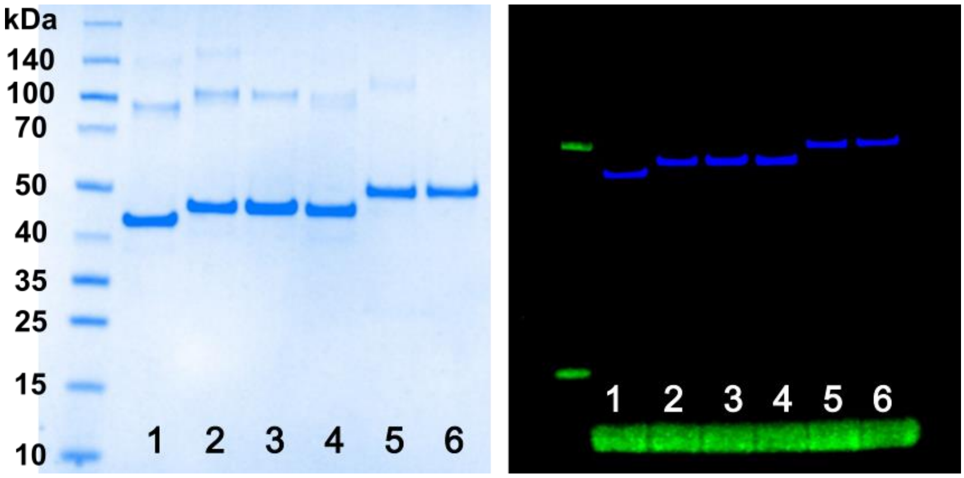

Fig. S1. SDS-PAGE analysis of purified LCD2-CTPR variants

A) Images of SDS-PAGE gels of purified LCD2-CTPR proteins. Left: Coomassie stained, Right: FlAsH labelled, 1: LCD2-CTPR2, 2: LCD2-CTPR3, 3: LCD2-R-CTPR3, 4: LCD2-CTPR4, 5: LCD2-CTPR4-ATG13, 6: LCD2-CTPR4-FUNDC1.

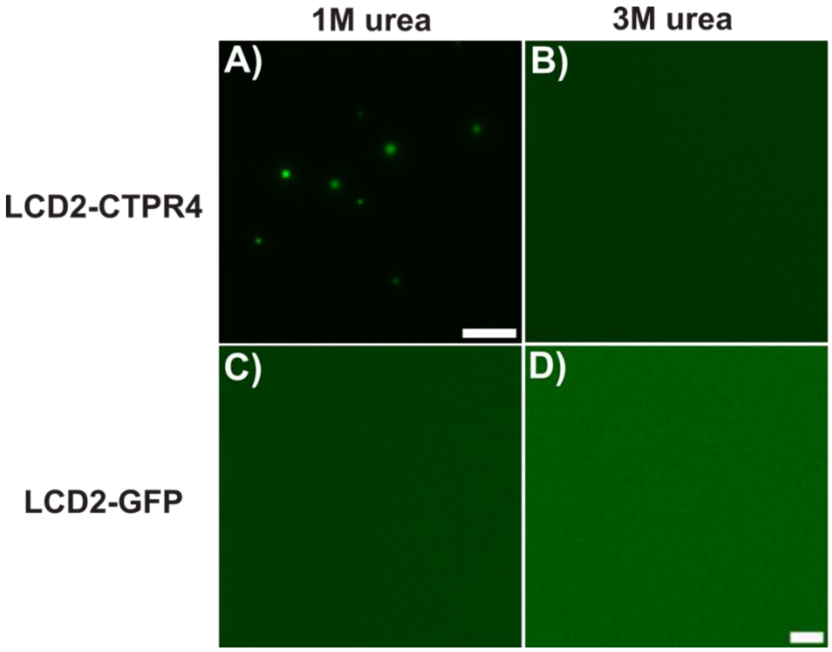

Fig. S2. Phase separation comparison between LCD2-CTPR4 and LCD2-GFP variants in different buffer conditions. Microscope images showing phase separation at 2 µM of (A, B) LCD1-CTPR4 and (C, D) LCD2-GFP in two different buffer conditions. (A, C) 50 mM Tris-HCl, pH 8.5, 1 M NaCl, 1 M urea, 0.5 mM TCEP. (B, D) 50 mM Tris-HCl, pH 8.5, 1 M NaCl, 3 M urea, 0.5 mM TCEP. Scale bar = 5 µm.

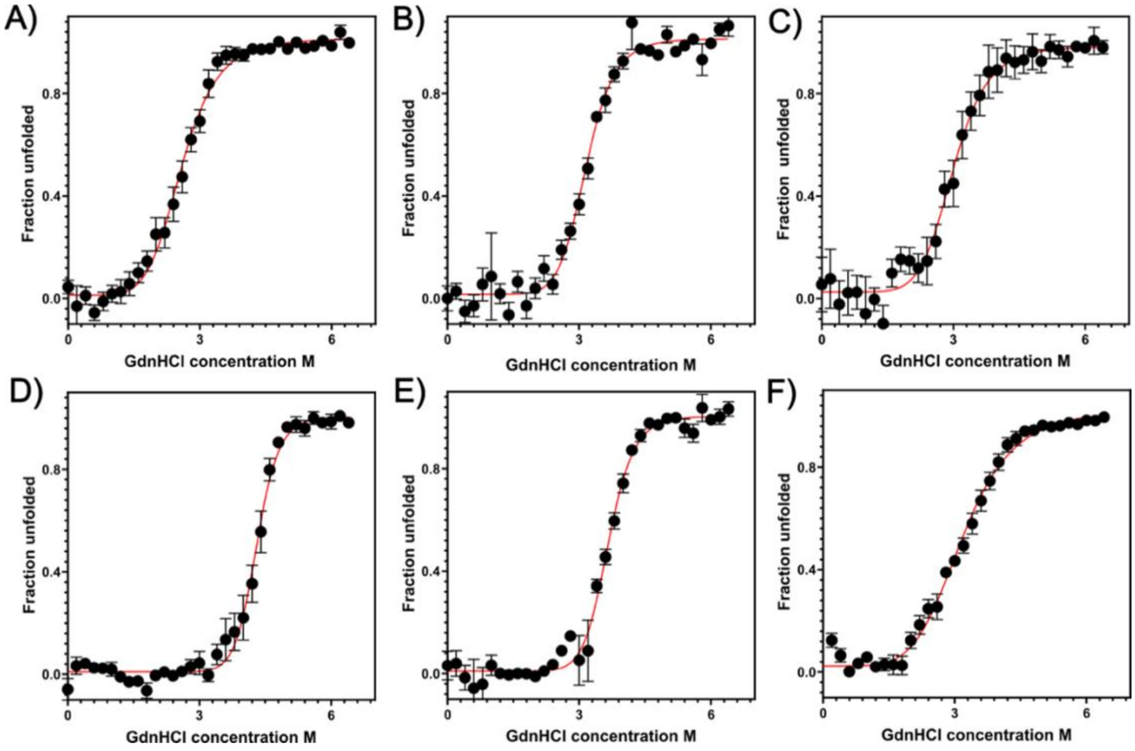

Fig. S3. GdnHCl-induced denaturation of CTPR-TC proteins. A) CTPR2, B) CTPR3, C) R-CTPR3, D) CTPR4, E) CTPR4-FUNDC1 and F) CTPR4-ATG13. 1 µm of protein in 50 mM

sodium phosphate, 150 mM NaCl, pH 7.5 with ranging GdnHCl concentration (from 0 M to 6.4 M) was excited at 295 nm at 25°C. Emission intensity was measured at 360 nm. A minimum of three independent experiments were recorded for each protein. The data were fitted with a two-state model to give the midpoint of unfolding and *m* value (a constant proportional to the change in solvent-accessible surface area upon unfolding). Error bars show the standard error of the mean.

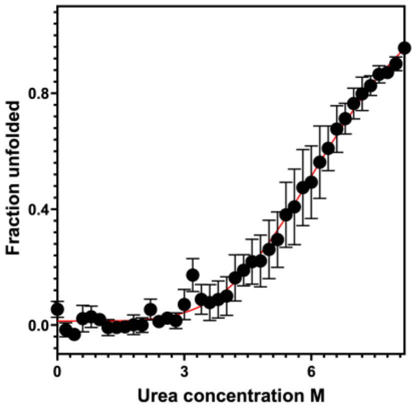

Fig. S4. Urea-induced denaturation of CTPR2. Urea-induced denaturation of CTPR2. 1 µm of protein in 50 mM sodium phosphate, 150 mM NaCl, pH 7.5 with urea concentration from 0 M to 8 M was excited at 295 nm at 25°C. Emission intensity was measured at 360 nm. Two independent experiments were recorded for this protein. The data were fitted with a two-state model to give the midpoint of unfolding and *m* value. Error bars show the standard error of the mean.

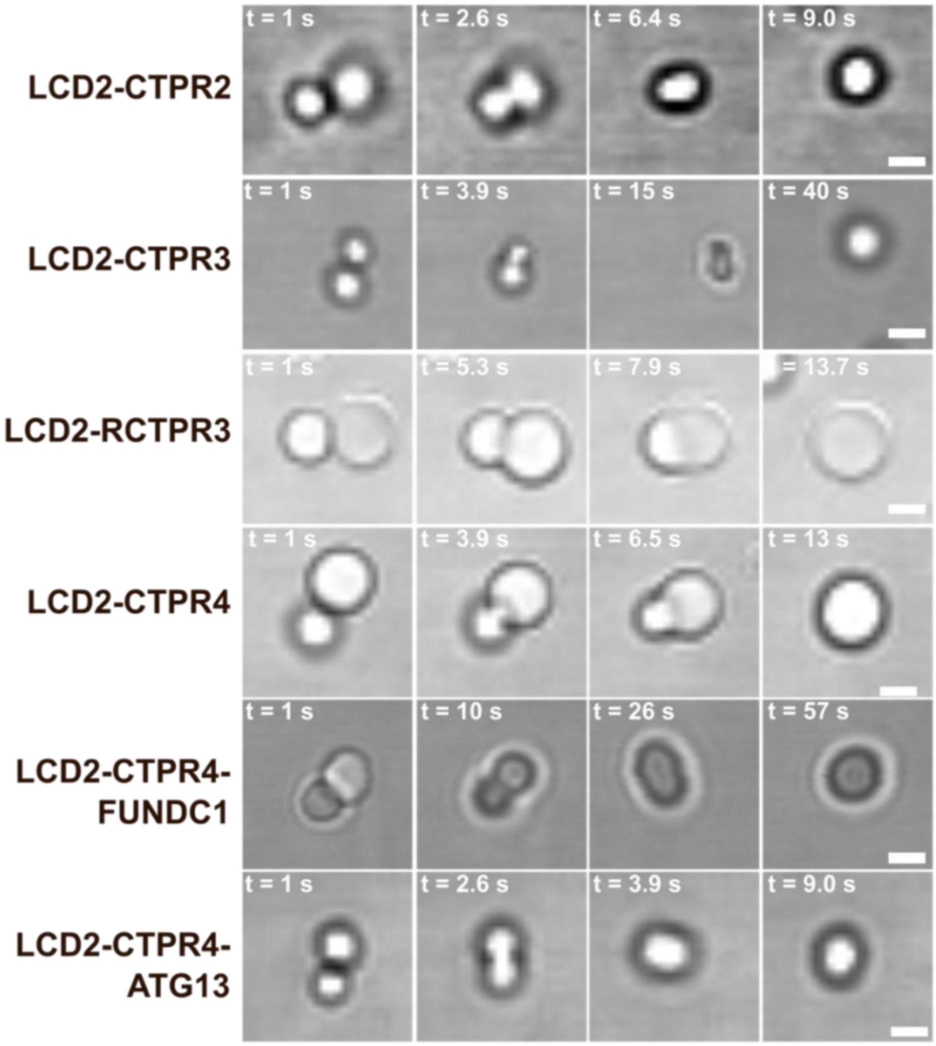

Fig. S5. Coalescence of the LCD2-CTPR variant condensates. 2 µM of each LCD2-CTPR variant was monitored in real-time in 50 mM Tris-HCl buffer at pH 8.5, 1000 mM NaCl. Scale bar is 2 µm.

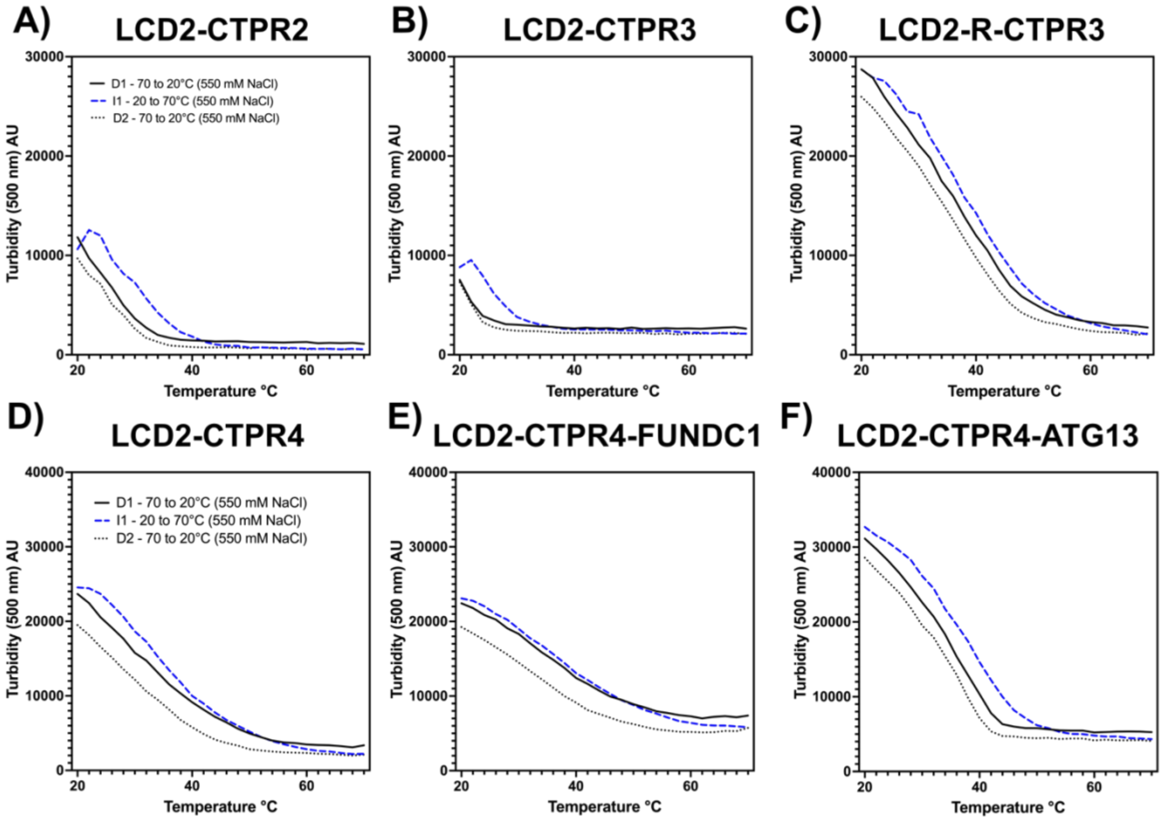

Fig. S6. Turbidity assay for LCD2-CTPR variants. Condensates were induced using standard dilution methods and heated to 70°C. The turbidity (at 500 nm) was monitored incrementally from 70 → 20°C (D1: solid black line), followed by an incremental increase from 20 → 70 °C (I1: dashed blue line) and finally another incremental decrease from 70 → 20°C (D2: dotted black line). Samples contained 2 µM protein, 50 mM Tris-HCl pH 7, 300 mM urea and 500 mM NaCl. Data is representative traces from n=3 independent experimental repeats.

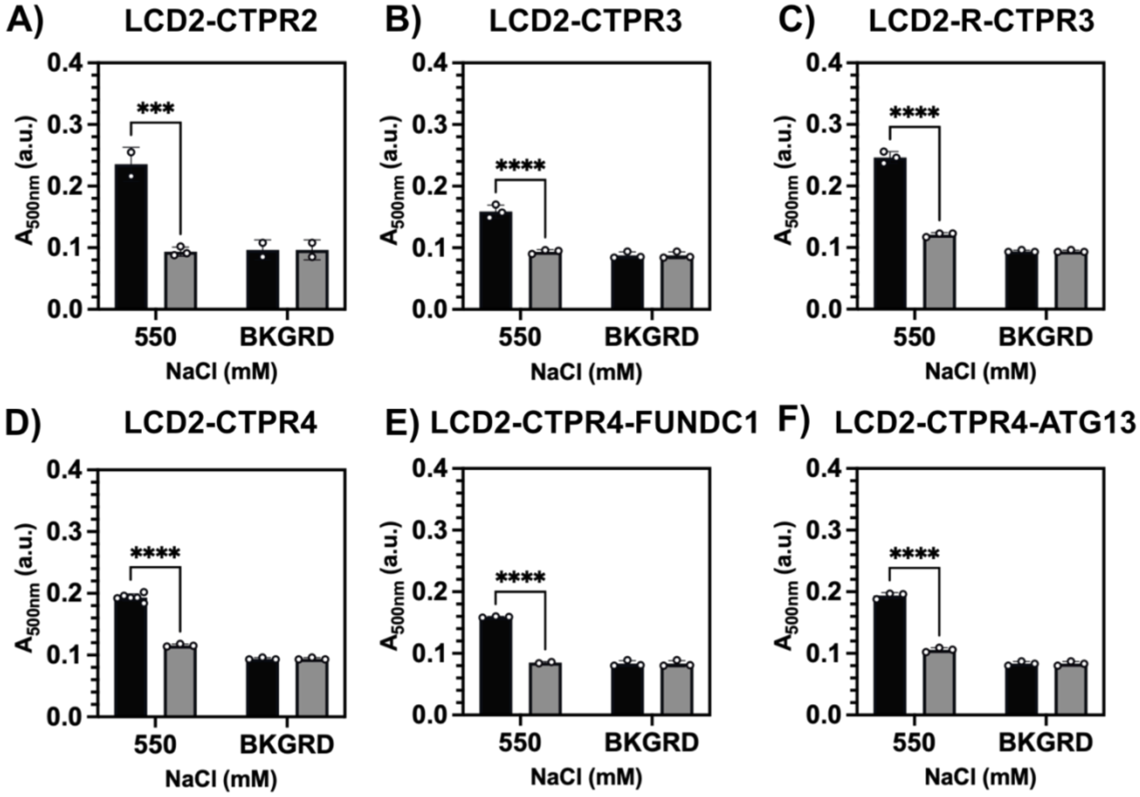

Fig. S7. 1,6-Hexanediol dissolution of LCD2-CTPRs constructs. Bar graphs (A-F) show the A_{500nm} for 2 μM LCD2-CTPR variants in 50 mM Tris-HCl, pH 7.0, with 550 mM NaCl, with (grey bars) and without (black bars) 4% 1,6-hexanediol (16H) treatment. Background absorbance is also shown as BKGRD. Significance determined by 2-way analysis of variance (ANOVA); **** $P < 0.0001$, *** $P < 0.0002$.

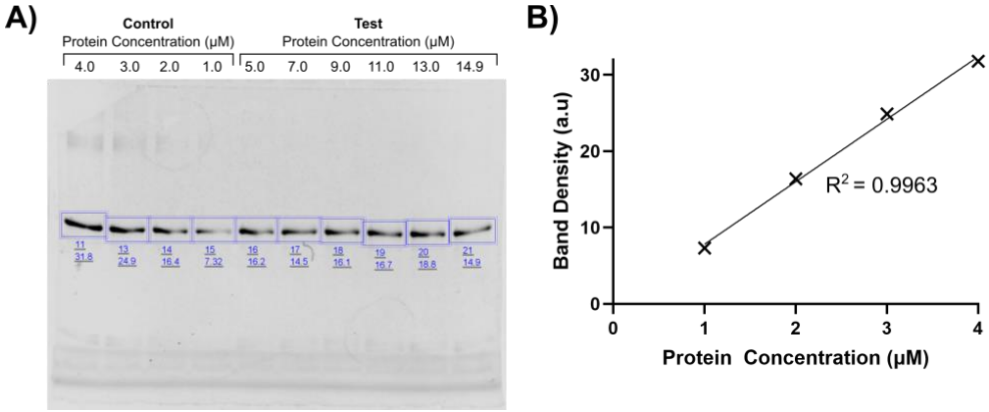

Fig. S8. Representative C_{Sat} assay method (for LCD2-CTPR2 at 550 mM NaCl). A) SDS-PAGE with quantified bands of FlAsH-labelled protein at varying total concentrations in control (50 mM Tris (pH 8.5), 1 M NaCl, 3 M Urea, 0.5 mM TCEP) or test (50 mM Tris (pH 7.0), 550 mM NaCl, 300 mM Urea, 0.5 mM TCEP) conditions. B) Linear regression of band density vs protein concentration in the control samples to generate standard curve for calculating concentration of test samples, R^2 = 0.9963.

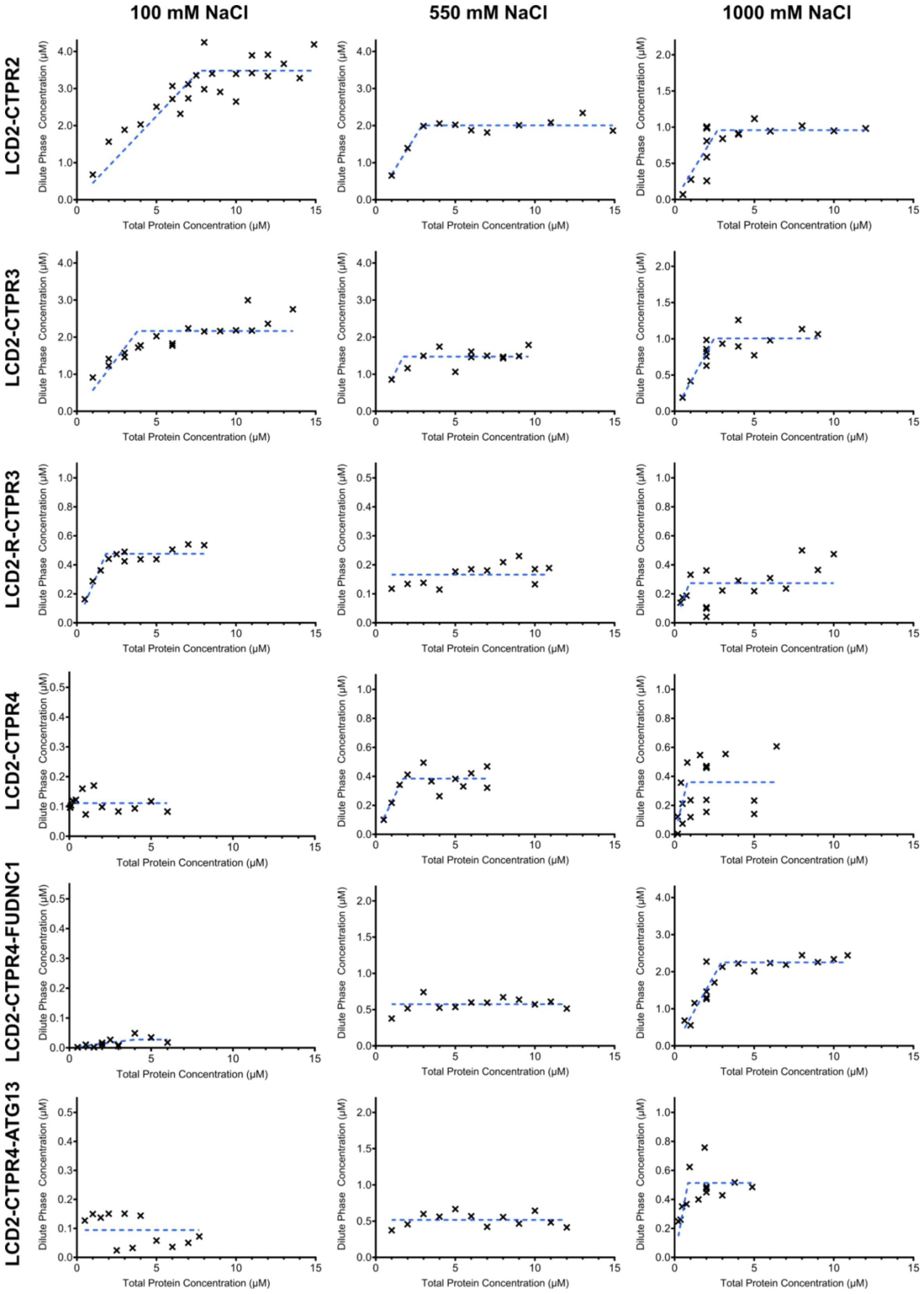

Fig. S9: C_{sat} assay for LCD2-CTPR variants. Plots show dilute phase concentrations reach plateau at C_{sat} concentration as total protein concentration increases. Proteins were in 50 mM Tris, pH 7, 300 mM urea, variable NaCl conditions.

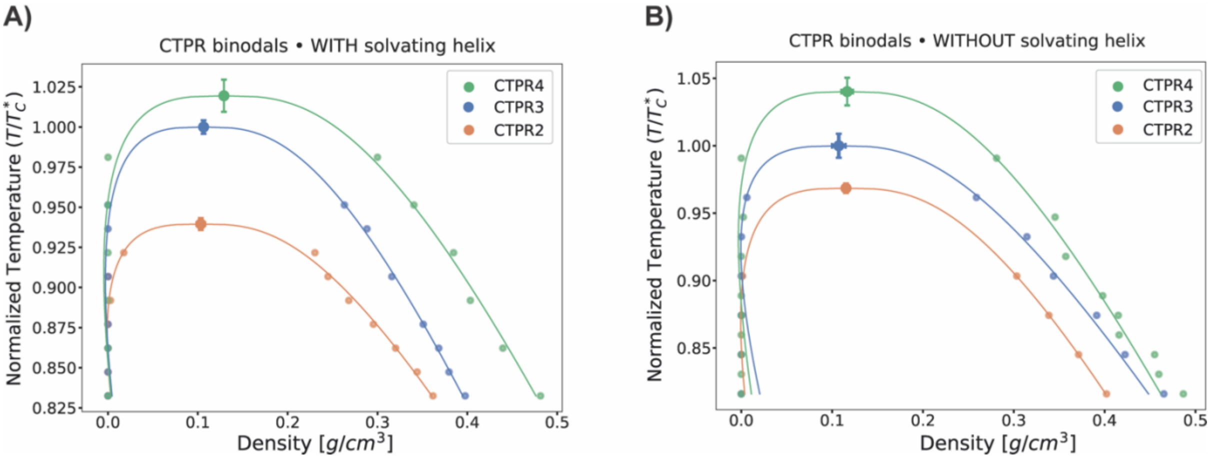

Fig. S10: *In silico* analysis of phase-separation of LCD2-CTPR variants in the presence or absence of a solvating helix. A) Regulation of critical parameters based on the number of CTPR repeats, all constructs with a solvating helix. Binodal in the Temperature-Density phase space of LCD2-CTPR2, LCD2-CTPR3 and LCD2-CTPR4-SH. Temperature on the y-axis is normalized by T*c, the critical temperature of LCD2-CTPR3-SH, to aid in comparison. B) Regulation of critical parameters based on the number of CTPR repeats, all constructs tested without a solvating helix. Binodal in the Temperature-Density phase space of LCD2-CTPR2 (no helix), LCD2-CTPR3 (no helix) and LCD2-CTPR4. Temperature on the y-axis is normalized by T*c, the critical temperature of LCD2-CTPR3 (no helix), to aid in comparison.

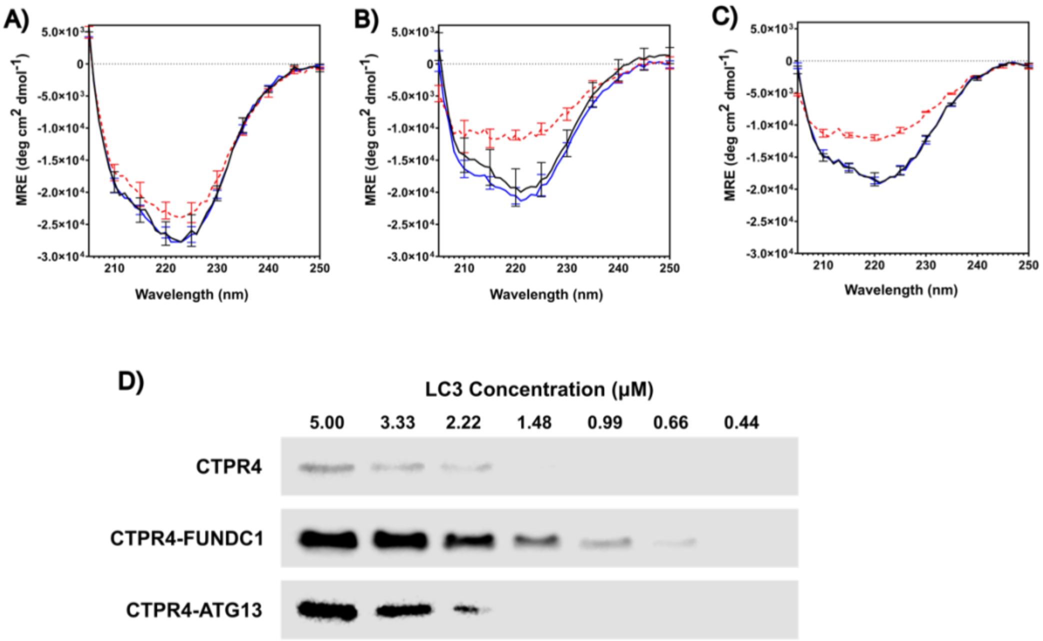

Fig. S11: A-C) CTPR-LIR Circular Dichroism characterisation. A) CTPR4, B) CTPR4-FUNDC1, C) CTPR4-ATG13. 5 µm of protein in 50 mM sodium phosphate, 150 mM NaCl, 0.5 mM TCEP, pH 7.5, spectra recorded from 205 to 250 nm in 0.5 nm intervals. Measured at 20°C (black), 90°C (red) and return to 20°C (blue). **D) CTPR-LIR – LC3 binding pull down.** Western blot showing the difference in capabilities of CTPR-LIR variants to pull down purified LC3 from solution at a range of concentrations. At high concentrations of LC3, we observe

some non-specific interactions with CTPR4 that we attribute to weak electrostatic interactions between the negatively charged surface of LC3 and positively charged residues in the CTPR protein.

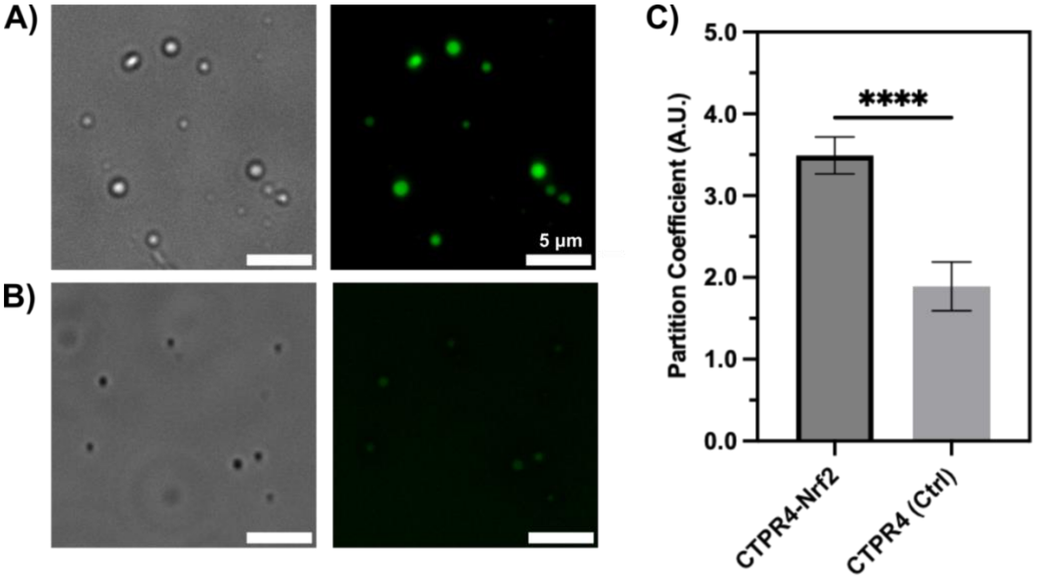

Fig S12. Recruitment of Keap1 into LCD2-RCTPR4-Nrf2 condensates. A) LCD2-RCTPR4-Nrf2 condensates (2 μM protein in 50 mM Tris-HCl (pH 7), 150 mM NaCl, 100 mM urea, 0.5 mM TCEP) incubated with Alexa488-labelled Keap1 (2 μM). Left: bright field, right: fluorescence. **B)** LCD2-CTPR4 condensates lacking the Nrf2 motif showed a lack of enrichment with Keap1. Scale bars: 5 μm. **C)** Partition coefficient quantification of Alexa488-labelled Keap1 for each LCD2-CTPR4 variant. Unpaired t-test used for statistical analysis; **** $P < 0.0001$.

Table S1. Amino acid sequences of LLPS-CTPR variants. The CTPR variant is indicated in bold and where substitutions to arginine are made, these are in blue italics. LCD1 N- and C-terminal sequences are in light blue, LCD2 N- and C-terminal sequences are in purple. Where applicable, the solvating helix is underlined. The tetracysteine-tag is indicated in green, the FUNDC1 sequence in red and the ATG13 sequence in blue. For optimised purification of full-length protein, LCD2-variants have an N-terminal His-tag, whereas LCD1-variants have a C-terminal His-tag.

Protein name	Amino acid sequence
LCD2-CTPR2	MRSGHHHHHHGLVPRGSADLPQKVSNLSINNKENGGGGGKSS<br>YVPPHLRSRGKPSFERRSPKQKDKVTGGDFFRRAGRQTGNNG<br>GFFGFSKERNGGTSANYNRRGSSNYKSSGNRWVNGKHIPGPK<br>NAKLQKAELFGVHDDPDYHSSGIKFDNYDNIPVDASGKDVPEPIL<br>GS **AEAWYNLGNAYYKQGDYQKAIEYYQKALELDPNN**CCPGCC<br>**DPNNAEAWYNLGNAYYKQGDYQKAIEYYQKALELDPRSAEAK<br>QNLGNAKQKQG**KLGGRTRGGGGFFNSRNNGSRDYRKHGGNG<br>SFGSTRPRNTGTSNWGSIGGGFRNDNEKNGYGNSNASWW
LCD2-CTPR3	MRSGHHHHHHGLVPRGSADLPQKVSNLSINNKENGGGGGKSS<br>YVPPHLRSRGKPSFERRSPKQKDKVTGGDFFRRAGRQTGNNG<br>GFFGFSKERNGGTSANYNRRGSSNYKSSGNRWVNGKHIPGPK

	NAKLQKAELFGVHDDPDYHSSGIKFDNYDNIPVDASGKDVPEPIL<br>GS **AEAWYNLGNAYYKQGDYQKAIEYYQKALELDPNN**CCPGCC<br>**DPNNAEAWYNLGNAYYKQGDYQKAIEYYQKALELDPRSAEAW**<br>**YNLGNAYYKQGDYQKAIEYYQKALELDPRS<u>AEAKQNLGNAKQ</u>**<br>**<u>KQG</u>**KLGGRTRGGGGFFNSRNNGSRDYRKHGGNGSFGSTRPR<br>NTGTSNWGSIGGGFRNDNEKNGYGNSNASWW
LCD2-R-CTPR3	MRSGHHHHHHGLVPRGSADLPQKVSNLSINNKENGGGGGKSS<br>YVPPHLRSRGKPSFERRSPKQKDKVTGGDFFRRAGRQTGNNG<br>GFFGFSKERNGGTSANYNRRGSSNYKSSGNRWVNGKHIPGPK<br>NAKLQKAELFGVHDDPDYHSSGIKFDNYDNIPVDASGKDVPEPIL<br>GS **AEAWYNLGNAYY*R*QGDYQ*R*AIEYYQ*R*ALELDPNN**CCPGCC<br>**DPNNAEAWYNLGNAYY*R*QGDYQ*R*AIEYYQ*R*ALELDPRSAEAW**<br>**YNLGNAYY*R*QGDYQ*R*AIEYYQ*R*ALELDPRS<u>AEAKQNLGNAKQ</u>**<br>**<u>KQG</u>**KLGGRTRGGGGFFNSRNNGSRDYRKHGGNGSFGSTRPR<br>NTGTSNWGSIGGGFRNDNEKNGYGNSNASWW
LCD2-CTPR4	MRSGHHHHHHGLVPRGSADLPQKVSNLSINNKENGGGGGKSS<br>YVPPHLRSRGKPSFERRSPKQKDKVTGGDFFRRAGRQTGNNG<br>GFFGFSKERNGGTSANYNRRGSSNYKSSGNRWVNGKHIPGPK<br>NAKLQKAELFGVHDDPDYHSSGIKFDNYDNIPVDASGKDVPEPIL<br>GS**AEAWYNLGNAYYKQGDYQKAIEYYQKALELDPNN**CCPGCC<br>**DPNNAEAWYNLGNAYYKQGDYQKAIEYYQKALELDPNNAEAW**<br>**YNLGNAYYKQGDYQKAIEYYQKALELDPNNAEAWYNLGNAYYK**<br>**QGDYQKAIEYYQKALELDPNN**KLGGRTRGGGGFFNSRNNGSRD<br>YRKHGGNGSFGSTRPRNTGTSNWGSIGGGFRNDNEKNGYGNS<br>NASWW
LCD2-CTPR4-<br>ATG13	MRSGHHHHHHGLVPRGSADLPQKVSNLSINNKENGGGGGKSS<br>YVPPHLRSRGKPSFERRSPKQKDKVTGGDFFRRAGRQTGNNG<br>GFFGFSKERNGGTSANYNRRGSSNYKSSGNRWVNGKHIPGPK<br>NAKLQKAELFGVHDDPDYHSSGIKFDNYDNIPVDASGKDVPEPIL<br>GS**AEAWYNLGNAYYKQGDYQKAIEYYQKALELDPNN**CCPGCC<br>**DPNNAEAWYNLGNAYYKQGDYQKAIEYYQKALELDPNNAEAW**<br>**YNLGNAYYKQGDYQKAIEYYQKALELDPNNGGSSGNTHDDFVM**<br>**IDFKPADPNNAEAWYNLGNAYYKQGDYQKAIEYYQKALELDPNN**<br>KLGGRTRGGGGFFNSRNNGSRDYRKHGGNGSFGSTRPRNTGTS<br>NWGSIGGGFRNDNEKNGYGNSNASWW
LCD2-CTPR4-<br>FUNDC1	MRSGHHHHHHGLVPRGSADLPQKVSNLSINNKENGGGGGKSS<br>YVPPHLRSRGKPSFERRSPKQKDKVTGGDFFRRAGRQTGNNG<br>GFFGFSKERNGGTSANYNRRGSSNYKSSGNRWVNGKHIPGPK<br>NAKLQKAELFGVHDDPDYHSSGIKFDNYDNIPVDASGKDVPEPIL<br>GS**AEAWYNLGNAYYKQGDYQKAIEYYQKALELDPNN**CCPGCC<br>**DPNNAEAWYNLGNAYYKQGDYQKAIEYYQKALELDPNNAEAW**<br>**YNLGNAYYKQGDYQKAIEYYQKALELDPNNDYESDDDSYEVLD**<br>**LTEYDPNNAEAWYNLGNAYYKQGDYQKAIEYYQKALELDPNN**<br>KLGGRTRGGGGFFNSRNNGSRDYRKHGGNGSFGSTRPRNTGT<br>SNWGSIGGGFRNDNEKNGYGNSNASWW
LCD2-RCTPR4-<br>Nrf2	MRSGHHHHHHGLVPRGSADLPQKVSNLSINNKENGGGGGKSS<br>YVPPHLRSRGKPSFERRSPKQKDKVTGGDFFRRAGRQTGNNG<br>GFFGFSKERNGGTSANYNRRGSSNYKSSGNRWVNGKHIPGPK<br>NAKLQKAELFGVHDDPDYHSSGIKFDNYDNIPVDASGKDVPEPIL<br>GS**AEAWYNLGNAYYRQGDYQRAIEYYQRALELDPNN**CCPGCC

	**DPNNAEAWYNLGNAYYRQGDYQRAIEYYQRALELDPNNAEAWYNLGNAYYRQGDYQRAIEYYQRALELDPNNLDPETGELLDPNNAEAWYNLGNAYYRQGDYQRAIEYYQRALELDPNN**KLGGRTRGGGGFFNSRNNGSRDYRKHGGNGSFGSTRPRNTGTSNWGSIGGGFRNDNEKNGYGNSNASWW

Table S2. Mass spectrometry results for purified LCD2-CTPR variants.

**Protein**	**Expected Mass /Da**	**Observed Mass /Da**
LCD2-CTPR2	36883.2	36886.5 ± 0.7
LCD2-CTPR3	40925.6	40928.3 ± 2.0
LCD2-RCTPR3	41177.7	41181.4 ± 6.3
LCD2-CTPR4	43356.1	43360.0 ± 5.3
LCD2-CTPR4-ATG13	45773.7	45777.0 ± 7.3
LCD2-CTPR4-FUNDC1	45849.6	45852.2 ± 1.7
LCD2-RCTPR4-Nrf2	45100.8	45105 ± 40 Da

Table S3. Parameters obtained from two-state fit of the GdnHCl-induced chemical denaturation experiments for CTPR variants lacking the molecular adhesives. All variants have a tetracysteine motif between repeats 1 and 2 unless stated otherwise. CTPR2, CTPR3, R-CTPR3 and CTPR3 (no TC-tag) all have a C-terminal solvating helix.

Protein	$D_{50\%}$ (M)	*m*-value (kcal mol^{-1} M^{-1})
CTPR2	2.6 ± 0.2	1.6 ± 0.4
CTPR3	3.0 ± 0.3	1.7 ± 0.6
R-CTPR3	3.1 ± 0.2	1.8 ± 0.9
CTPR4	4.3 ± 0.2	2.8 ± 0.9
CTPR4-ATG13	3.3 ± 0.3	1.5 ± 1.1
CTPR4-FUNDC1	3.6 ± 0.1	2.1 ± 0.5
CTPR3 (no TC-tag)	3.6 ± 0.03	2.7 ± 0.5
CTPR4 (no TC-tag)	4.8 ± 0.1	3.8 ± 0.6

Uncropped gels and western blots

Supplementary Figure 1:

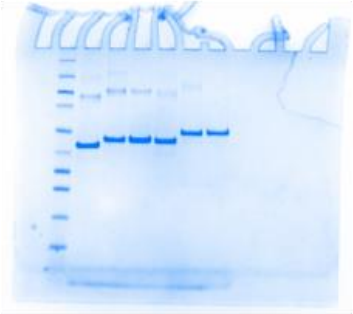

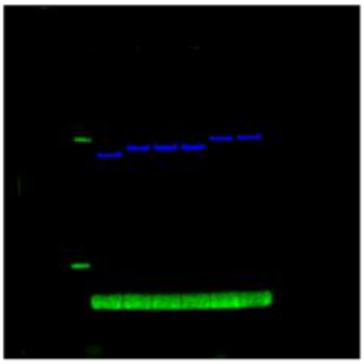

Supplementary Figure 9:

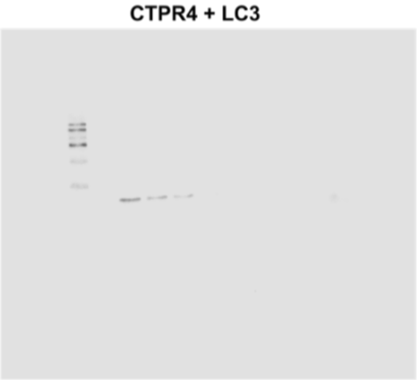

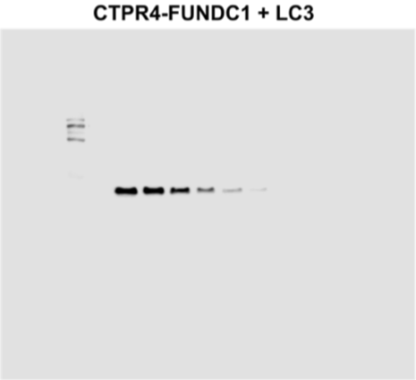

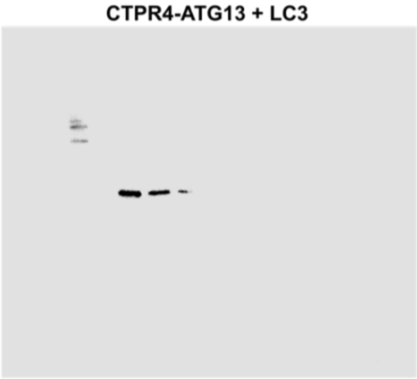

Supplement: SC-016-D5SC00903K-s001 [file SC-016-D5SC00903K-s001.pdf]
